# A Review of Bayesian Perspectives on Sample Size Derivation for Confirmatory Trials

**DOI:** 10.1080/00031305.2021.1901782

**Published:** 2021-04-22

**Authors:** Kevin Kunzmann, Michael J. Grayling, Kim May Lee, David S. Robertson, Kaspar Rufibach, James M. S. Wason

**Affiliations:** a MRC Biostatistics Unit, University of Cambridge, Cambridge, UK; b Population Health Sciences Institute, Newcastle University, Newcastle upon Tyne, UK; c Pragmatic Clinical Trials Unit, Queen Mary University of London, London, UK; d Methods, Collaboration, and Outreach Group (MCO), Department of Biostatistics, F. Hoffmann-La Roche, Basel

**Keywords:** Assurance, Expected power, Probability of success, Power, Sample size derivation

## Abstract

Sample size derivation is a crucial element of planning any confirmatory trial. The required sample size is typically derived based on constraints on the maximal acceptable Type I error rate and minimal desired power. Power depends on the unknown true effect and tends to be calculated either for the smallest relevant effect or a likely point alternative. The former might be problematic if the minimal relevant effect is close to the null, thus requiring an excessively large sample size, while the latter is dubious since it does not account for the a priori uncertainty about the likely alternative effect. A Bayesian perspective on sample size derivation for a frequentist trial can reconcile arguments about the relative a priori plausibility of alternative effects with ideas based on the relevance of effect sizes. Many suggestions as to how such “hybrid” approaches could be implemented in practice have been put forward. However, key quantities are often defined in subtly different ways in the literature. Starting from the traditional entirely frequentist approach to sample size derivation, we derive consistent definitions for the most commonly used hybrid quantities and highlight connections, before discussing and demonstrating their use in sample size derivation for clinical trials.

## 1 Introduction

Randomized controlled trials (RCTs) are the gold-standard study design for evaluating the effectiveness and safety of new interventions. However, the real-world proportion of RCTs is low (Wong, Siah, and Lo 2019), which negatively affects the cost of drug development (DiMasi, Grabowski, and Hansen 2016). The sample size of a trial is a key determinant of both cost and the chances of detecting a treatment effect (if it is present). Purely economic arguments would suggest a utility based approach as discussed in, for example, Lindley (1997). In practice, the specification of a utility function for a clinical trial is often impeded by the difficulty of quantifying ethical considerations and compliance with health authority guidelines. For instance, an RCT with an unnecessarily large sample size (“overpowered”) would be unethical if the treatment showed a substantial effect and the consequences of being randomized to the control arm were severe. Too small an RCT (“underpowered”) would also be unethical if it resulted in future patients being deprived access to an effective treatment due to a substantial effect going undetected. Consequently, the majority of RCTs derive their sample size based on desired Type I and Type II error rates.

The traditional approach to determining the sample size for a trial is to choose a point alternative and derive a sample size such that the probability to reject the null hypothesis exceeds a certain threshold (typically 80% or 90%) while maintaining a specified maximal Type I error rate (typically 2.5% one-sided). The maximal Type I error rate is usually realized at the boundary of the null hypothesis and can thus be computed without further assumptions. The Type II error rate, however, critically depends on the choice of the (point) alternative for which there are at least two ways of justifying its choice. The first is based on a *relevance* argument, which requires the specification of a minimal clinically relevant difference (MCID). The probability to reject the null hypothesis is typically monotonic in the effect size and consequently the power for all other relevant differences will be even larger than under the MCID. For guidance on the choice of the MCID see, for example, Cook et al. (2018). The second perspective is based on a priori considerations about the likelihood of the treatment effect. Here, an a priori likely effect is used as the point alternative (typically larger than the MCID) implying that the resulting sample size might be too small to detect smaller but still relevant differences reliably although the potential savings in terms of sample size might still outweigh the risk of ending up with an underpowered study. The core difference between these approaches is that a MCID-based sample size is not subject to uncertainty since the MCID is generally considered fixed based on relevance arguments. In contrast, choosing the point alternative based on considerations about the relative a priori likelihood of effect sizes implies that there is an inherent uncertainty about the effect size, and thus the required sample size—otherwise no trial would be needed in the first place.

Other approaches to sample size calculation which are beyond the scope of this article may target a certain width of the confidence interval for the AUC (Obuchowski 1998), or the standard error of an estimate (Grouin et al. 2007; Thompson 2012). Group-sequential or adaptive trial designs which allow a trial to be stopped early if the observed effect is much smaller or larger than anticipated are another way to cope with a priori uncertainty about the effect size at the planning stage (Jennison and Turnbull 2000; Bauer et al. 2016; Wassmer and Brannath 2016).

Consider the case of a one-stage, one-arm *Z*-test (see Section 5 for a two-arm trial example) where the interest lies in testing the null hypothesis 𝓗_0_ : *θ* ≤ *θ*_0_ = 0 at a one-sided significance level of *α*. Let *X_i_*, *i* = 1, . . . , *n*, be iid observations with mean *θ* and known standard deviation *σ*. Under suitable regularity conditions, the mean is asymptotically normal and $Z_{n}:=\sqrt{n}\left({\bar{X}}_{n}-\theta_{0}\right)/\sigma \dot{∼}N\left(\theta_{n},1\right)$, where ${\bar{X}}_{n}:=1/n\sum_{i=1}^{n}X_{i}$ is the sample mean and $\theta_{n}:=\sqrt{n}\left(\theta -\theta_{0}\right)/\sigma$. The critical value for rejecting 𝓗_0_ is given by the (1 − *α*)-quantile of the standard normal distribution, *z*_1−*α*_, and is independent of *n*. The probability of rejecting the null hypothesis for given *n* and *θ* is

(1) $${\mathrm{Pr}}_{\theta }\left[Z_{n}>z_{1-\alpha }\right]=1-\text{Φ}\left(z_{1-\alpha }-\theta_{n}\right)=\text{Φ}\left(\theta_{n}-z_{1-\alpha }\right),$$

where Φ is the cumulative distribution function (CDF) of the standard normal distribution. Often, Pr_*θ*_[*Z_n_* > *z*_1−*α*_]is seen as a function of *θ* and termed the “power function.” This terminology may lead to confusion when considering parameter values *θ* ≤ *θ*_0_ and *θ* ≥ *θ*_MCID_, since the probability to reject the null hypothesis corresponds to the Type I error rate in the former case and classical ”power” in the latter. For the sake of clarity we therefore use the neutral term “probability to reject.”

Assume that a point alternative *θ*_alt_ > *θ*_0_ is given. A sample size can then be chosen as the solution of

(2) $$\begin{array}{lll} n_{\theta_{\text{alt}\;}}^{*}:= & \underset{n}{\text{argmin}}:n & \\ & \text{subject}\;\text{to}: & {\text{Pr}}_{\theta_{\text{alt}\;}}\left[Z_{n}>z_{1-\alpha }\right]\geq 1-\beta . \end{array}$$

Since Pr_*θ*_[ *Z_n_* > *z*_1−*α*_] is monotone in *θ*, Pr_*θ*_[ *Z_n_* > *z*_1−*α*_] ≥ 1 − *β* ∀*θ* ≥ *θ*_alt_ and if *θ*_alt_ = *θ*_MCID_, the null hypothesis can be rejected for all clinically relevant effects with a probability of at least 1 − *β*. This approach requires no assumptions about the a priori likelihood of the value of *θ* but only about *θ*_MCID_ and the desired minimal power (see also Chuang-Stein 2006; Chuang-Stein et al. 2011, sec. 3). However, the required sample size increases quickly as *θ*_MCID_ approaches *θ*_0_. The problem is aggravated if the null hypothesis is defined as ${ℋ}_{0}^{\prime }:\theta \leq$ and *θ*_MCID_ > 0, that is, if the primary study objective is to demonstrate a clinically important effect. In either case it is impossible to derive a feasible sample size based on the MCID alone (Chuang-Stein et al. 2011). Due to the difficulties of eliciting a sample size in such situations, in practice, trialists may resort to back-calculating an effect size in order to achieve the desired power given the maximum feasible sample size (Lenth 2001;Grouin et al. 2007; Lan and Wittes 2012). One way of justifying a smaller sample size is to simply consider a *likely* point alternative *θ*_alt_ > *θ*_MCID_ instead. This pragmatic approach is unsatisfactory since it ignores any uncertainty about the assumed effect (Lenth 2001).

In the following, we first review approaches to quantifying the probability to reject the null hypothesis when a prior distribution is available. Wherever necessary, we refine existing definitions to improve overall consistency. We then discuss their application to sample size calculation. We exclusively focus on what is termed a “hybrid” Bayesian-frequentist approach (Spiegelhalter, Abrams, and Myles 2004). This means that, although Bayesian arguments are used to derive a sample size under uncertainty about the true effect, the final analysis is strictly frequentist. A structured overview of all quantities considered is provided in Figure 2. We present a review of the literature on the subject in the supplemental material, showcasing the confusing diversity of terminology used in the field and relating our definitions back to the existing literature. Finally, we apply the methods to a clinical trial example and conclude with a discussion.

## 2 Bayesian Assessment of the Probability to Reject the Null Hypothesis

One way of incorporating planning uncertainty is to make assumptions about the relative a priori likelihood of the unknown effect size. This approach can be formalized within a Bayesian framework by seeing the true effect *θ* as the realization of a random variable Θ with prior density *φ*(*θ*). At the planning stage, the probability to reject the null hypothesis is then given by the random variable RPR(*n*) := Pr_Θ_[ *Z_n_* > *z*_1−*α*_] (“random probability to reject”). We explicitly denote this quantity as “random” to emphasize the distinction between the (conditional on Θ = *θ*) probability to reject given in Equation (1) and the unconditional “random” probability to reject. The variation of the random variable RPR(*n*) reflects the a priori uncertainty about the unknown underlying effect that is encoded in the prior density *φ*(·). We define the random variable “random power” as RPow(*n*) := Pr_Θ≥*θ*_MCID__ [*Z_n_* > *z*_1−*α*_]. Note that RPow(*n*) = RPR(*n*)|Θ ≥ *θ*_MCID_. The distribution of either the (unconditional) random probability to reject the null hypothesis or the (conditional) random power can then be used to define summary measures. We discuss some options in the following.

### 2.1 A Prior Quantile-based Approach

Spiegelhalter and Freedman (1986) note that a power constraint for sample size derivation could be computed based on “[...] a somewhat arbitrarily chosen location parameter of the [prior] distribution (for example the mean, the median or the 70th percentile).” This essentially means that the prior uncertainty is collapsed by choosing a suitable location parameter of the prior distribution of Θ for *θ*_alt_. Using a location parameter of the unconditional prior distribution to assess the rejection probability, however, is difficult to interpret when the chosen location parameter lies within the null hypothesis (i.e., for skeptical prior distributions). Instead, we follow a similar idea but motivate the choice of location parameter in terms of the a priori distribution of random power and thus conditional on a relevant effect. Let Q_*p*_[*Y*] denote the *p*-quantile of the random variable *Y*.Then

(3) $${\text{Q}}_{1-\gamma }[\text{RPow}(n)]=\underset{x}{\inf}\;{\mathrm{Pr}}_{\varphi (\cdot )}[\text{RPow}(n)\geq x]\geq \gamma$$

is the (1 − *γ*)-quantile of the random power.^1^ The probability to reject is a monotone function in *θ*. Hence,

(4) $$\begin{array}{ll} {\text{Q}}_{1-\gamma }[\text{RPow}(n)] & ={\text{Q}}_{1-\gamma }\left[\mathrm{Pr}\text{Θ}\geq \theta_{\text{MCID}}\left[Z_{n}>z_{1-\alpha }\right]\right]\\ & ={\mathrm{Pr}}_{{\text{Q}}_{1-\gamma }\left[\text{Θ}|\text{Θ}\geq \theta_{\text{MCID}}\right]}\left[Z_{n}>z_{1-\alpha }\right]. \end{array}$$

Reducing random power to a certain quantile of its distribution is thus equivalent to evaluating the probability to reject at the corresponding quantile of the *conditional* prior distribution. Other than with Spiegelhalter and Friedmann’s unconditional approach, who addressed the issue of the location parameter potentially falling within the null hypothesis by using unconditional *p*-quantiles with sufficiently large *p*, any quantile of the conditional prior distribution is guaranteed to be larger than *θ*_MCID_. The quantile approach is practically appealing since it reduces to justifying the choice of *θ*_alt_ in a Bayesian way. However, it is complicated by the need to choose the additional parameter *γ* .

### 2.2 Probability of Success

Spiegelhalter and Freedman (1986) also proposed the use of the “probability of concluding that the new treatment is superior and of this being correct (*P_Ss_* in their notation) to derive a required sample size. The quantity has subsequently been referred to as “prior adjusted power” (Spiegelhalter, Abrams, and Myles 2004; Shao,Mukhi, and Goldberg 2008), and is also discussed in Liu (2010) and Ciarleglio et al. (2015). In the situation at hand, it is

(5) $$\text{PoS}(n):=\mathrm{Pr}\left[Z_{n}>z_{1-\alpha },\text{Θ}\geq \theta_{\text{MCID}}\right]$$

(6) $$={\int^{\text{​}}}_{\theta_{\text{MCID}}}^{\infty }{\int^{\text{​}}}_{z_{1-\alpha }}^{\infty }\phi \left(z-\theta_{n}\right)\varphi (\theta )\;\text{d}\;z\;\text{d}\;\theta ,$$

where *ϕ* is the probability density function (PDF) of the standard normal distribution. Here, we are more general than previous authors in that we allow *θ*_MCID_ > 0 and use a tighter definition of “success”: a trial is only successful if the null hypothesis is rejected *and* the effect is relevant. Whenever *θ*_MCID_ = 0 this coincides with the definitions used previously in the literature.

The definition of PoS(*n*) critically relies on what is considered a “success”. Spiegelhalter and Freedman only considered a significant result a success if the underlying effect is also nonnull (i.e., the joint probability of nonnull *and* detection). More recently, a majority of authors tend to follow O’Hagan et al. who define the probability of success by integrating the probability to reject over the entire parameter range (O’Hagan and Stevens 2001; O’Hagan, Stevens, and Campbell 2005) and term this “assurance”. For a more comprehensive overview of the terms used in the literature, see Section B in the supplemental material. The alternative definition for probability of success introduced by O’Hagan et al. corresponds to the marginal probability of rejecting the null hypothesis irrespective of the corresponding parameter value

(7) $${\text{PoS}}^{\prime }(n):=\mathrm{Pr}\left[Z_{n}>z_{1-\alpha }\right]$$

(8) $$={\int^{\text{​}}}_{-\infty }^{\infty }{\int^{\text{​}}}_{z_{1-\alpha }}^{\infty }\phi \left(z-\theta_{n}\right)\varphi (\theta )\;\text{d}\;z\;\text{d}\;\theta$$

(9) $$\begin{array}{ll} =\text{PoS}(n) & \\ & +\underset{\text{probability}\;\text{of}\;\text{rejection}\;\text{and}\;\text{irrelevant}\;\text{effect}\;}{\underset{︸}{\mathrm{Pr}\left[Z_{n}>z_{1-\alpha },0<\text{Θ}<\theta_{\text{MCID}}\right]}}\\ & +\underset{\text{probability}\;\text{of}\;\text{a}\;\text{Type}\;\text{I}\;\text{error}\;}{\underset{︸}{\mathrm{Pr}\left[Z_{n}>z_{1-\alpha },\text{Θ}\leq 0\right]}}. \end{array}$$

The decomposition in Equation (9) shows that the implicit definition of “success” underlying PoS′(*n*) is at least questionable (Liu 2010). The marginal probability of rejecting the null hypothesis includes rejections under irrelevant or even null values of *θ* . This issue was first raised by Spiegelhalter, Abrams, and Myles (2004)for point null and alternative hypotheses. For more practically relevant scenarios with prior mean greater than *θ*_0_ = 0 and *θ*_MCID_ ≈ *θ*_0_, the contribution of the average Type I error rate to PoS′(*n*) is almost negligible (see supplemental material, Section A). If *θ*_MCID_ = 0, the numeric difference between PoS and PoS′ is negligible since the maximal Type I error rate is controlled at level *α* and the power curve quickly approaches zero on the interior of the null hypothesis. Spiegelhalter, Abrams, and Myles (2004) thus argued that PoS′(*n*) can be used as an approximation to PoS(*n*) in many (but not all) practically relevant situations.

Which definition of “success” is preferred depends on perspective: a short-term oriented pharmaceutical company may just be interested in rejecting the null hypothesis to monetize a new drug, irrespective of it actually showing a relevant effect. This view would correspond to PoS′(*n*). Regulators and companies worried about the longer term consequences ofpotentially having to retract ineffective drugs, may tend toward the joint probability of correctly rejecting the null. We take the latter perspective and focus on PoS(*n*).

### 2.3 Expected Power

Probability ofsuccess is an unconditional quantity and therefore depends on the a priori probability ofa relevant effect

(10) $$\text{PoS}(n)=\mathrm{Pr}\left[Z_{n}>z_{1-\alpha },\text{Θ}\geq \theta_{\text{MCID}}\right]$$

(11) $$={\int^{\text{​}}}_{\theta_{\text{MCD}}}^{\infty }{\mathrm{Pr}}_{\theta }\left[Z_{n}>z_{1-\alpha }\right]\varphi (\theta )\text{d}\theta$$

(12) $$=\mathrm{Pr}\left[Z_{n}>z_{1-\alpha }|\text{Θ}\geq \theta_{\text{MCID}}\right]\mathrm{Pr}\left[\text{Θ}\geq \theta_{\text{MCID}}\right]$$

(13) $$\begin{array}{ll} =\underset{=\text{E}[\text{RPow}(n)]=:\text{EP}(n)}{\underset{︸}{\text{E}\left[{\mathrm{Pr}}_{\text{Θ}\geq \theta_{\text{MCD}}}\left[Z_{n}>z_{1-\alpha }\right]\right]}} & \mathrm{Pr}\left[\text{Θ}\geq \theta_{\text{MCID}}\right] \end{array}.$$

This means that PoS(*n*) can be expressed as the product of the “expected power”, EP(*n*), and the a priori probability of a relevant effect (see again Spiegelhalter, Abrams, and Myles 2004 for the situation with point hypotheses). Expected power was implicitly mentioned in Spiegelhalter and Freedman (1986) (*P_Ss_*/*P*_·*s*_ in their notation) as a way to characterize the properties of a design.

Unfortunately, the terms “expected power’‘ and “probability of success” are sometimes used interchangeably in the literature (see supplemental material Section B). Expected power is merely a weighted average of the probability to reject in the relevance region *θ* ≥ *θ*_MCID_, where the weight is given by the conditional prior density

(14) $$\begin{array}{ll} \varphi \left(\theta |\text{Θ}\geq \theta_{\text{MCID}}\right) & :=\varphi (\theta )1_{\theta \geq \theta_{\text{MCID}}}{\left({\int^{\text{​}}}_{\theta_{\text{MCID}}}^{\infty }\varphi (y)\text{d}y\right)}^{-1} \end{array},$$

which means

(15) $$\begin{array}{ll} \text{EP}(n) & {\int^{\text{​}}}_{\theta_{\text{MCID}}}^{\infty }{\mathrm{Pr}}_{\theta }\left[Z_{n}>z_{1-\alpha }\right]\varphi \left(\theta |\text{Θ}\geq \theta_{\text{MCID}}\right)\text{d}\theta \end{array}.$$

PoS(*n*), on the other hand, integrates the probability to reject over the same region using the unconditional prior density (see Equations (11) and 15)). Thus, in contrast to PoS(*n*), expected power does not depend on the a priori probability of a relevant effect but only on the relative magnitude of the prior density (“a priori likelihood”) of relevant parameter values. Since the conditional prior density differs from the unconditional one only by normalization via the a priori probability of a relevant effect, it follows from Equation (13) that EP(*n*) and PoS(*n*) differ only by the constant factor Pr[Θ ≥ *θ*_MCID_ ].

Comparing expected power to a quantile of the random power (see Section 2.1), an advantage lies in the fact that no additional parameter *γ* needs to be specified. However, unlike the quantile approach (compare Equation (4)), forming the expected value cannot simply be interchanged with the nonlinear probability to reject:

(16) $$\text{E}[\text{RPow}(n)]\neq {\mathrm{Pr}}_{\text{E}\left[\text{Θ}|\text{Θ}\geq \theta_{\text{MCID}}\right]}\left[Z_{n}>z_{1-\alpha }\right].$$

The probability to reject at the prior expected effect given that the effect is relevant is thus different from “expected power.”

## 3 Prior Choice

A major issue in the Bayesian modeling of uncertainty is the elicitation of an adequate prior. As illustrated in Rufibach, Burger, and Abt (2016), the prior crucially impacts the properties and interpretability of any Bayesian functional of a design’s power curve. Often, there is no direct prior knowledge on the effect size of interest. Researchers are then often tempted to use a *vague* prior, typically a normal prior with large variance, as, for example, advocated in Saint-Hilary et al. (2019). Assuming a non-informative, improper prior for Θ would imply that arbitrarily large effect sizes are just as likely as small ones. Yet, in clinical trials,the standardized effect size rarely exceeds 0.5 (Lamberink et al. 2018). We thus illustrate the characteristics of the different approaches to defining power constraints under uncertainty using a convenient truncated Gaussian prior. The truncated Gaussian is conjugate to a Gaussian likelihood and allows us to restrict the plausible range of effect sizes to, for example, liberally [−1, 1]. Also, the truncated Gaussian is the maximum entropy distribution on the truncation interval, for a given mean and variance, which can be interpreted as a”least-informative” property under constraints on the first two moments. Prior elicitation is also discussed in Spiegelhalter, Abrams, and Myles (2004). A more formal prior elicitation framework is SHELF (Kinnersley and Day 2013; Oakley and O’Hagan 2019) and Dallow, Best, and Montague (2018) discusses how SHELF is routinely used by pharmaceutical companies.

## 4 Application to Sample Size Calculation

Any functional of a design’s power curve that depends monotonically on *n* can be used to derive a sample size by imposing a (w.l.o.g.) lower boundary on its value. For the classical frequentist approach to sample size calculation, this functional is the probability to reject the null hypothesis at *θ*_alt_ or at *θ*_MCID_.

Using expected power as the functional, let $n_{\text{EP}}^{*}$ be the smallest *n* that satisfies EP (*n*) ≥ 1 − *β*. The power function is monotonically increasing in *θ* and thus expected power is strictly larger than power at the minimal relevant value whenever Pr[Θ > *Θ*_MCID_ ] > 0. This implies a constraint on expected power is less restrictive than a constraint on the probability to reject the null hypothesis at *θ*_MCID_. Consequently, for the same threshold 1 − *β*, the required sample size under an expected power constraint is smaller. Since expected power and probability of success differ only by a constant factor, any constraint on EP(*n*) can be transformed to a corresponding constraint on PoS(*n*)

(17) $$\text{PoS}(n)\geq 1-\beta \Leftrightarrow \text{EP}(n)\geq (1-\beta )/\mathrm{Pr}\left[\text{Θ}\geq \theta_{\text{MCID}}\right]$$

Furthermore, PoS(*n*) = EP(*n*)Pr[Θ ≥ *θ*_MCID_ ] and EP(*n*) ≤ 1, thus PoS(*n*) can never exceed the a priori probability of a relevant effect. This implies the usual conventions on the choice of *β* as the maximal Type II error rate for a point alternative cannot be meaningful in terms of the unconditional PoS(*n*), since the maximum attainable probability of success is the situation-specific a priori probability of a relevant effect. The need to recalibrate typical benchmark thresholds when considering probability of success was previously discussed in the literature. For instance, O’Hagan, Stevens, and Campbell (2005) state that “[t]he assurance figure is often much lower [than the power], because there is an appreciable prior probability that the treatment difference is less than δ*”, where δ* corresponds to *θ*_MCID_ in our notation. A similar argument is put forward in Rufibach, Burger, and Abt (2016, Section 2) for PoS′(*n*). The key issue is thus whether one is interested in the joint probability of rejecting the null hypothesis *and* the effect being relevant, PoS(*n*), or the conditional probability of rejecting the null hypothesis *given* a relevant effect, EP(*n*).

To make the difference between EP(*n*) and PoS(*n*) for sample size calculation more tangible, consider a situation in which the a priori probability of Θ ≥ *θ*_MCID_ is 0.51. The probability of success is then only 41% (for 80% expected power) or 46% (for 90% expected power). A sponsor might want to increase these relatively low unconditional success probabilities by deriving a sample size based on a minimal PoS(*n*) of 1 − *β* instead. The choice of 1 − *β* is limited by the a priori probability of a relevant effect (0.51 in this case). Using Equation (17) a minimal probability of success of 0.5 is equivalent to requiring an expected power of more than 98%. In essence, the attempt to increase PoS(*n*) via a more stringent threshold on EP(*n*) implies that low a priori chances of success are to be offset with almost certain detection (EP(*n*) ≈ 1) in the unlikely event of an effect actually being present.

Alternatively, let $n_{\text{EP}}^{*}$ be the smallest *n* that satisfies Q_1−*γ*_ [ RPow(*n*)] ≥ 1 − *β*. By definition, this implies that the a priori probability of exceeding a probability to reject of 1 − *β* given a relevant effect would be at least *γ*. Since Pr_*θ*_[ *Z_n_* > *z*_1−*α*_] is monotonic in *θ*, this problem is equivalent to finding the smallest *n* that satisfies Pr _Q_1−*γ*_ [Θ≥*θ*_MCID_ ]_[*Z_n_* > *z*_1−*α*_] ≥ 1 − *β*. Consequently, this “prior quantile approach” can be used with any existing frequentist sample size formula. It is merely a formal Bayesian justification for determining the sample size of a trial based on a point alternative *θ*_alt_ := Q_1−*γ*_ [Θ ≥ *θ*_MCID_ ] ≥ *θ*_MCID_ and reduces to powering on *θ*_MCID_ whenever the target power needs to be met with absolute certainty for all relevant effects (*γ* = 1).

### 4.1 Required Sample Sizes for Various Prior Choices

**Figure 1:**
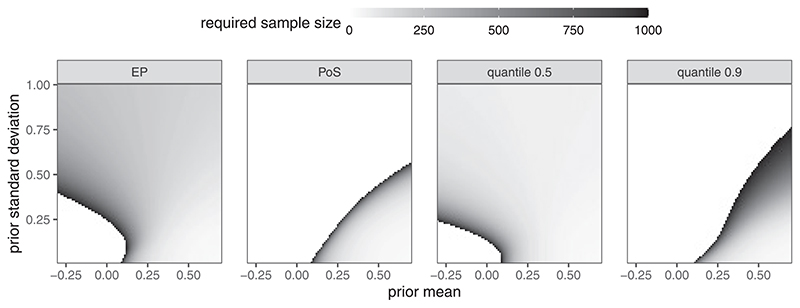
Required sample size plotted against prior parameters (Normal truncated to [−0.3, 0.7], with varying mean and standard deviation); *θ*_MCID_ = 0.1; EP = Expected Power, PoS = Probability of Success, quantile = quantile approach with *γ* = 0.5 and *γ* = 0.9, respectively.

Let *θ*_MCID_ = 0.1 and the maximal feasible sample size be 1000. Figure 1 shows the required sample sizes under the expected power, probability of success, and quantile approaches (*γ* = 0.5, 0.9). We use *α* = 0.025 and 1 − *β* = 0.8 for all methods.

For probability of success, large prior uncertainty implies low a priori probability of a relevant effect and thus the required sample sizes explode for large prior standard deviations (in relation to the prior mean). For very large standard deviations, the constraint on probability of success becomes infeasible (white area). The expected power criterion leads to a completely different sample size pattern. Since expected power is defined conditional on a relevant effect, large prior uncertainty increases the weight in the upper tails of the power curve where power quickly approaches one. Consequently, for small prior means, larger uncertainty decreases the required sample size. For large prior means, however, smaller prior uncertainty leads to smaller sample sizes since again more weight is concentrated in the tails of the power curve. The characteristics of the prior-quantile approach very much depend on the choice of *γ*. When using the conditional prior median (*γ* = 0.5) the approach is qualitatively similar to the expected power approach. This is due to the fact that computing power on the conditional median of the prior is close to computing power on the conditional prior mean.

Since the power function is locally linear around the center of mass of the conditional prior, this approximates computing expected power by interchanging forming the expected value and computing power (i.e., first average the prior and then compute power or average over power with weights given by the conditional prior). For a stricter criterion (*γ* = 0.9) the required sample sizes are much larger. Higher uncertainty then decreases the (1−*γ* )-quantile toward the minimal relevant effect and thus increases the required sample size.

### 4.2 Connection to Utility Maximization

In a regulatory environment, and most scientific fields, the choice of *α* is a pre-determined quality criterion. Yet, the exact choice of the threshold 1 − *β* is much more arbitrary. In clinical trials, 1 − *β* = 0.9 or 1 − *β* = 0.8 are common choices when a classical sample size derivation is conducted. From the previous section, it is clear a threshold 1 − *β* which is independent of the specific context of a trial only makes sense when using conditional quantities like the probability to reject at a conditional prior quantile or EP(*n*) to derive a required sample size.

Unconditional measures such as PoS(*n*) tend to be easier to interpret and arise naturally in the context of utility maximization or maximal expected utility (MEU). An in-depth discussion of the MEU concept is beyond the scope of this article and we refer the reader to, for example, Lindley (1997) or Lai (1984) for a discussion of utility considerations in a sequential setting. We focus on highlighting the fact that the choice of the constraint threshold 1 − *β* can be justified by making the link to MEU principles. This merely formalizes arguments discussed in a classical sensitivity analysis where the final sample size or power is fixed. In particular, the final power might deviate from the default 80% or 90% depending on the effect on sample size and thus costs.

Assume that the maximal Type I error rate is still to be controlled at level *α*. For sake of simplicity, further assume that a *correct* rejection of the null hypothesis yields an expected return of *λ* (in terms of the average per-patient costs within the trial). Ignoring fixed costs, the expected trial utility is then

(18) U(n):=λPoS(n)−n.

The utility-maximizing sample size is $n_{U}^{*}(\lambda ):={\text{argmax}}_{n}U(n)$. The same *n* would be obtained when determining the sample size based on expected power if the threshold $1-\beta =PoS\left(n_{U}^{*}(\lambda )\right)/\mathrm{Pr}\left[\text{Θ}\geq \theta_{\text{MCID}}\right]=EP\left(n_{U}^{*}(\lambda )\right)$ was used. Similarly, one could start with $n_{\text{EP}}^{*}$ for a given *β* and derive the corresponding *λ* such that $n_{U}^{*}(\lambda )=n_{\text{EP}}^{*}$. This value of *λ* would then correspond to the implied expected reward upon successful rejection of the null for given *β*. Under the assumption of a utility function of the form (18), *λ* and *β* can thus be matched such that the corresponding utility maximization problem and the constraint minimization of the sample size under a power constraint both lead to the same required sample size. Consequently, practitioners are free to either define an expected return upon successful rejection, *λ*, or a threshold on the minimal expected power, 1 − *β*. We give a practical example of this process in Section 5.

## 5 A Clinical Trial Example

Consider the case of a clinical trial designed to demonstrate superiority of an intervention over a control with respect to the hazard ratio of overall survival. The required sample size for a log-rank test can be derived under the assumption of proportional hazards. Let *n* be the overall sample size across both treatment arms, η the anticipated proportion of study participants dying within the follow up time of the study, and the hazard ratio of the intervention relative to the control arm. The log-rank test *z*-statistic is asymptotically normally distributed with mean $-\log(\xi )\sqrt{\eta \;n/4}$ and standard deviation 1 (Schoenfeld 1981). Here, the sign implies that *ξ* < 1 (superiority of the intervention) corresponds to larger *Z*-scores. For the sake of simplicity, we further assume that *η* = 0.33 is known although one could also assume a prior distribution over this nuisance parameter. Up to the constant factor $\sqrt{\eta /4}$ this setting corresponds to the previously discussed one-arm *Z*-test if we define *θ* := −log(*ξ* ).

Let the prior for the treatment effect on the log hazard ratio scale be given by a truncated Normal distribution on [−log(1.5), −log(0.5)] ≈ [−0.41, 0.69] with mean 0.2 and standard deviation 0.2 (pre-truncation). The corresponding prior density on the hazard ratio scale is given in the left panel of Figure 3. The MCID is set to *θ*_MCID_ = 0.05 which corresponds to a hazard ratio of approximately 0.95. In this setting the a priori probability of a relevant effect is approximately 0.86. Figure 3 shows the prior density on the hazard ratio scale, the curves of the rejection probability corresponding to the required sample sizes derived from constraints on a minimal probability to reject of 1 − *β* = 0.8 at *θ*_MCID_ (MCID), at Q_0.5_[Θ ≥ *θ*_MCID_ ] ≈ 0.26 (quantile, 0.5, hazard ratio: 0.77), at Q_0.9_[Θ ≥ *θ*_MCID_ ] ≈ 0.10 (quantile, 0.9, hazard ratio: 0.91), or a minimal expected power of 1 − *β* = 0.8 (EP), as well as the CDFs of the corresponding distribution of random power.

The MCID criterion requires *n* = 35,799. The quantile approach (with *γ* = 0.9) reduces this to *n* = 9806 while still maintaining an a priori chance of 90% to exceed the target power of 80%. The quantile approach with *γ* = 0.5 results in the lowest sample size of *n* = 1434 at the cost of only having a 50% chance to exceed the target power of 80%. The EP approach is more liberal than the quantile approach (*γ* = 0.9) with *n* = 2588 but still guarantees a chance of exceeding the target power of roughly 65% (Figure 3, right panel). A sample size based on PoS(*n*) ≥ 1 − *β* = 0.8 cannot be derived in this example since the a priori probability of a relevant effect is 0.78, lower than 0.8. The large spread between the derived sample sizes shows how sensitive the required sample size is to the changes in the power constraint. Clearly, the MCID approach is highly inefficient, as accepting a small chance to undershoot the target power with the quantile approach (*γ* = 0.9) already reduces the required sample size by more than two thirds (from *n* = 35799 to *n* = 9806). At the other extreme, constraining power on the conditional prior median (quantile approach, *γ* = 0.5) leads to a rather unattractive a priori distribution of the random power: by definition, the probability to exceed a rejection probability of 0.8 is still 0.5 but the a priori chance of ending up with a severely underpowered study is non-negligible (long left tail of the CDF in the right panel of Figure 3). The corresponding unconditional success probabilities (PoS) are: 0.77 for MCID, 0.62 for EP, 0.73 for the quantile 0.9, and 0.53 for the quantile 0.5 approach.

These considerations leave the trial sponsor with essentially two options. One option is to consider a range of scenarios for the quantile approach with values of *γ* between 0.5 and 0.9 in more detail. A decision on the exact value of *γ* could be reached by considering the corresponding distributions of RPow(*n*). Alternatively, the intermediate EP approach could be used. The required sample size for an (expected) power of 80% is *n* = 2588. Note that with this option, there is an implicit tradeoff inherent to expected power: there is a roughly one-in-five a priori probability to end up in a situation with less than 50% power (see Figure 3, right panel).

In a situation where 1 − *β* = 0.8 is not set in stone, further insights may be gained by making the link to utility maximization explicit. One may consider that the sponsor has no way of quantifying the reward parameter *λ* directly. Decision making is then guided by mapping the threshold on expected power to the implied reward *λ* as discussed in Section 4.2. Figure 4 shows this “implied reward” as a function of the minimal expected power constraint. An expected power of 0.8 is achieved ifthe expected reward upon successful (i.e., the effect is indeed relevant) rejection of the null hypothesis is approximately 20,489 times the average per-patient costs within the planned trial. Using the curve depicted in Figure 4, the plausibility of certain reward levels can be discussed with the trial sponsor. When the average per-patient costs are well-known in advance, the scale can be transformed to monetary units. For example, consider the expected average per-patient costs are 30,000 $US. The sample size corresponding to an expected power of 0.8 is maximizing the utility if the expected reward is 30, 000 × 20, 489 = 61.85 × 10^7^ $US. The utility-maximizing reward for an expected power of 0.9 would be approximately 70,534, that is, 211.60 ·10^7^ $US. Even without committing to a fixed value of *λ*, these considerations can be used to guide the decision as to which of the “standard” power levels (0.8 or 0.9) might be more appropriate in the situation at hand.

Of course, one might also directly optimize utility if the reward upon successful rejection of the null hypothesis can be specified. To that end, assume that a reward of 30 × 10^7^ $US is expected. Under the same assumption about average per-patient costs, this translates to *λ* ≈ 10,000. The utility-maximizing sample size is then *n* = 1590 and the corresponding utilitymaximizing expected power is 0.71.

## 6 Discussion

**Figure 2:**
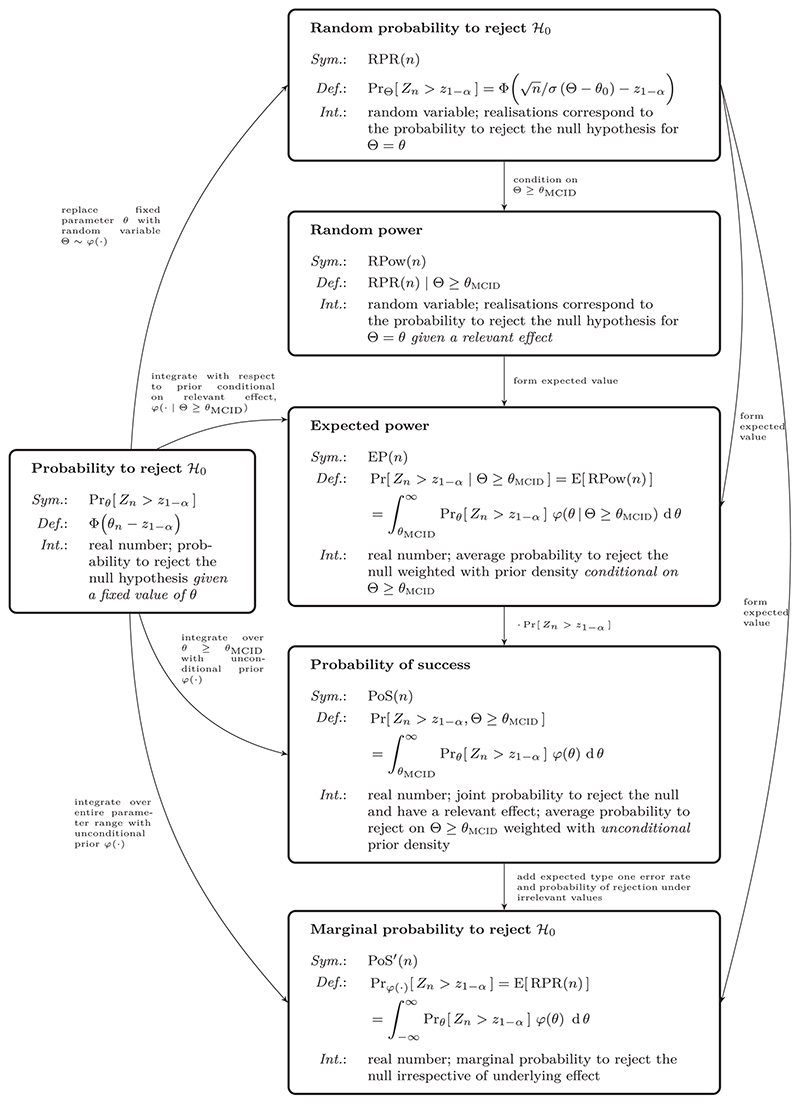
Structured overview of all quantities related to “power”that are introduced in Section 1 to 2.2. The symbols used in the text (*Sym*.), their exact definitions (*Def*.), and verbal interpretation (*Int*.) are summarized in the respective boxes. The relationships between the individual quantities are given as labeled arrows. For a structured overview of previous mentions and synonyms used in the literature, see Table 1 in the supplemental material.

The concept of “hybrid” sample size derivations are well-established in the literature on clinical trial design. Nevertheless, the substantial variation in the terminology used and small differences in their exact definitions can be confusing. Our contribution here is to highlight connections between different quantities and provide a naming scheme using definitive terminologies (see Figure 2). Any naming scheme necessarily has a subjective element to it and ours is by no means exempt from this problem (see also https://xkcd.com/927/). We encourage a clearer separation between terminology for joint probabilities (avoiding the use of the word “power”) and for probabilities that condition on the presence of an effect. An explicit definition (in formulae) of any quantities used should be given when discussing the subject. Referring to terms like “expected power” or “probability of success” are too ambiguous given their inconsistent historical use.

A hybrid approach to sample size derivation can incorporate the uncertainty about the true underlying effect into the design. This approach allows a natural distinction between arguments relating to the (relative) a priori likelihood of different parameter values (encoded in the prior density) and relevance arguments (encoded in the choice of *θ*_MCID_). The fact that these components can be represented naturally within the hybrid approach has the potential to make sample size derivation more transparent.

The hybrid quantity considered most commonly in the literature is the marginal probability to reject 𝓗_0_. It is important to appreciate that this quantity includes the error of rejecting the null hypothesis incorrectly. In many practical situations this problem is numerically negligible and PoS′(*n*) ≈ PoS(*n*). If, however, the definition of “success” also takes into account a non-trivial relevance threshold *θ*_MCID_ > *θ*_0_, the distinction becomes more important. Given the great emphasis on strict Type i error rate control in the clinical trials community it seems at least strange to implicitly consider Type I errors as “successful” trial outcomes.

While EP(*n*) is independent of the a priori probability of a relevant effect and only depends on the relative a priori likelihood of different effects through the conditional prior, PoS(*n*) does depend on Pr[ Θ ≥ *θ*_MCID_ ]. Although Spiegelhalter, Abrams, and Myles (2004), see this as a disadvantage of EP(*n*), it is actually a necessary property to use it for sample size derivation without recalibrating the conventional values for 1 − *β* (see also Brown et al. 1987). Unconditional quantities like PoS(*n*) do, however, play a key role in utility maximization approaches (see Section 4.2) and in communicating the risks associated with the conduct of a study.

**Figure 3:**
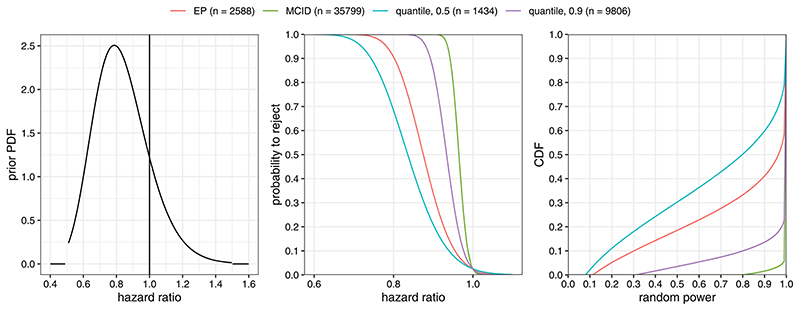
Left panel: prior PDF; middle panel: probability to reject the null hypothesis as function of the hazard ratio exp (−*θ*); right panel: CDF of randompower (probability to reject given Θ > *θ*_MCID_ = 0.05) for the four different design choices.

**Figure 4:**
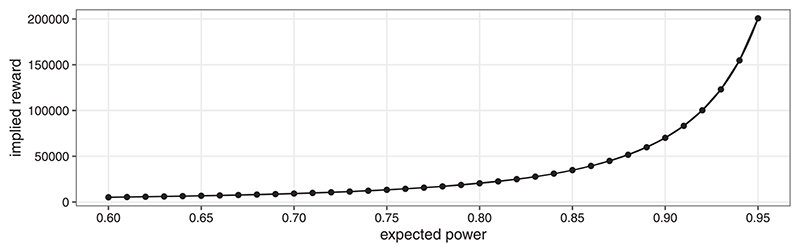
Example of utility-maximizing implied reward *λ* for varying expected power.

The “quantile approach” is an alternative concept to sample size calculation, which uses a different functional of the probability to reject the null hypothesis given a relevant effect. It considers a Bayesian justification for powering on a particular point alternative and is thus easy to implement. Instead of the mean, we propose to use a (1 − *γ* ) quantile of this distribution. Compared to expected power, this allows direct control of the left-tail of the a priori distribution of the probability to reject the null hypothesis given a relevant effect. Controlling the lower tail of the power distribution explicitly can be desirable since a sample size derived via a threshold for expected power might still lead to a substantial chance of ending up with an underpowered study. This flexibility comes at the price of having to specify an additional parameter, *γ* . To choose between the expected power and the prior quantile approach, it is advisable to not only plot the corresponding power curves but also the resulting distribution of RPow(*n*) (see Figure 3).

Finally, it should be stressed again that the key frequentist property of strict Type I error rate control is not affected by the fact that the arguments for calculating a required sample size are Bayesian. In fact, at no point, is Bayes theorem is invoked. The Bayesian perspective is merely a principled and insightful way of specifying a weight function (prior density) that can be used to guide the choice of the power level of the design, or as Brown et al. (1987, p. 30) put it: “This proposed use of Bayesian methods should not be criticized by frequentists in that these methods do not replace any current statistical techniques, but instead offer additional guidance where current practice is mute.”

## Supplementary Material

Supplementary File Code to reproduce all figures is available at https://github.com/kkmann/sample-size-calculation-under-uncertainty/tree/0.3.0 and permanentlybacked up to zenodo.org (Kunzmann et al. 2020).A non-interactive version of the Jupyter notebook used to create the figures for this publication is available at https://github.com/kkmann/sample-size-calculation-underuncertainty/blob/0.3.0/sample-size-calculation-under-uncertainty.ipynb An interactive version of the repository at the time of publication is hosted using mybinderhub.org and Binder (Jupyter et al. 2018) at https://mybinder.org/v2/gh/kkmann/sample-size-calculation-under-uncertainty/0.3.0?urlpath=lab/tree/sample-size-calculation-under-uncertainty.ipynb.
A shiny app implementing the sample size calculation procedures is available at https://mybinder.org/v2/gh/kkmann/sample-size-calculationunder-uncertainty/0.3.0?urlpath=shiny/shiny-app/. The interactive services are made available free of charge and thus only provide limited performance; the startup of the interactive link and the shiny app may take up to 2 minutes.

## Footnotes

1 Here, we choose to make the dependency of Pr_*φ*(·)_ RPow(*n*) ≥ *x* on the prior density explicit by using the index “*φ*(·)” since the random variable Θ does not appear directly in the description of the event “RPow(*n*) ≥ *x*.” We omit the index “*φ*” whenever Θ appears explicitly.
