# Supplementary material for: A Review of Bayesian Perspectives on Sample Size Derivation for Confirmatory Trials
Source: Am Stat. Author manuscript; Available in PMC 2022 Jan 5. (PMC7612172; doi:10.1080/00031305.2021.1901782)
Supplement: Supplementary File — Code to reproduce all figures is available at https://github.com/kkmann/sample-size-calculation-under-uncertainty/tree/0.3.0 and permanentlybacked up to zenodo.org (Kunzmann et al. 2020).A non-interactive version of the Jupyter notebook used to create the figures for this publication is available at https://github.com/kkmann/sample-size-calculation-underuncertainty/blob/0.3.0/sample-size-calculation-under-uncertainty.ipynb An interactive version of the repository at the time of publication is hosted using mybinderhub.org and Binder (Jupyter et al. 2018) at https://mybinder.org/v2/gh/kkmann/sample-size-calculation-under-uncertainty/0.3.0?urlpath=lab/tree/sample-size-calculation-under-uncertainty.ipynb. A shiny app implementing the sample size calculation procedures is available at https://mybinder.org/v2/gh/kkmann/sample-size-calculationunder-uncertainty/0.3.0?urlpath=shiny/shiny-app/. The interactive services are made available free of charge and thus only provide limited performance; the startup of the interactive link and the shiny app may take up to 2 minutes. [file EMS140634-supplement-Supplementary_File.pdf]

Supplemental Material to “A Review of Bayesian Perspectives on Sample Size Derivation for Confirmatory Trials”

Kevin Kunzmann MRC Biostatistics Unit
University of Cambridge
East Forvie Site, Robinson Way, Cambridge Biomedical Campus
Cambridge CB2 0SR, United Kingdom
kevin.kunzmann@mrc-bsu.cam.ac.uk
Michael J. Grayling
Population Health Sciences Institute
Newcastle University
michael.grayling@newcastle.ac.uk
Kim May Lee
Pragmatic Clinical Trials Unit
Queen Mary University of London
k.m.lee@qmul.ac.uk
David S. Robertson
MRC Biostatisics Unit
University of Cambridge
david.robertson@mrc-bsu.cam.ac.uk
Kaspar Rufibach
Methods, Collaboration, and Outreach Group (MCO)
Department of Biostatistics
F. Hoffmann-La Roche, Basel
kaspar.rufibach@roche.com
James M. S. Wason
Population Health Sciences Institute
Newcastle University
and
MRC Biostatistics Unit
University of Cambridge
james.wason@newcastle.ac.uk

March 8, 2021

A Sensitivity of Probability of Success with respect to the definition of “success”

The degree to which $\mathrm{PoS}(n)$ and $\mathrm{PoS}'(n)$ differ numerically is visualized in Figure 1. It depicts the proportion of the individual components of $\mathrm{PoS}'(n)$ for varying prior standard deviation and prior means. The sample size is fixed at $n = 150$, $\theta_0 = 0$, the maximal type I error rate is $\alpha = 0.025$, and the minimal clinically important difference is $\theta_{\text{MCID}} = 0.1$. A truncated normal prior on $[-1, 1]$ with varying mean and standard deviation was used. The contribution of type I errors (component “A” in Figure 1) to $\mathrm{PoS}'(n)$ is mostly negligible unless the prior is sharply peaked at an effect size slightly smaller than the null. The *a priori* probability of a relevant effect size is close to zero in these cases and so is $\mathrm{PoS}'(n)$.

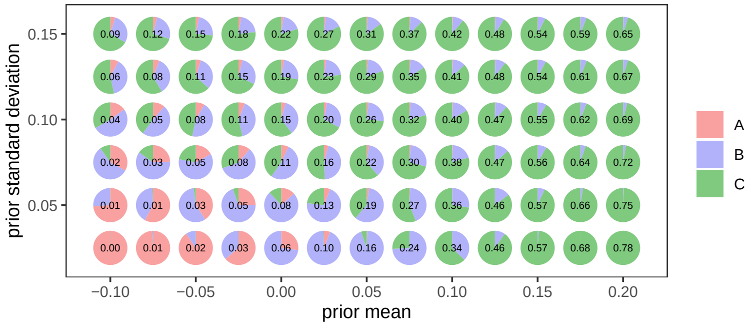

Figure 1: Components of $\mathrm{PoS}'(n)$ for $n = 150$, $\theta_0 = 0$, $\alpha = 0.025$, $\theta_{\text{MCID}} = 0.1$ and varying prior mean and standard deviation; numbers correspond to overall $\mathrm{PoS}'(n)$; proportions in individual pie charts correspond to: A = probability to reject and null effect (type I error), B = probability to reject and irrelevant but non-null effect, C = probability to reject and relevant effect (PoS).

B Literature review of terminology

A structured overview of the literature on “hybrid” Bayesian sample size derivation in the context of clinical trials is given in Table 1. The table relates publications in the field to the terms defined in Figure 2 of the main text. Publications with a similar take on the matter are grouped. In the following, we highlight a few particularly interesting contributions and how they relate to the definitions used in this manuscript.

The majority of the manuscripts only consider the marginal probability to reject $\mathcal{H}_0$ ($\text{PoS}'(n)$). Many publications refer to O'Hagan and Stevens (2001) or O'Hagan *et al.* (2005), where this quantity was introduced as "assurance". The range of names for what we call the "marginal probability to reject $\mathcal{H}_0$" is, however, quite diverse: "assurance", "probability of success", "predictive probability of success", "average probability of success", "probability of statistical success", "probability of study success", "predictive power", "predictive frequentist power", "expected power", "average power", "strength", "extended Bayesian expected power 1", and "hybrid Neyman-Pearson-Bayesian probability".

However, only a handful of authors elaborate on the intricacies of defining what exactly constitutes a "success" and whether to consider an unconditional measure of success or to condition on the presence of a relevant effect for sample size derivation (Spiegelhalter and Freedman, 1986; Brown *et al.*, 1987; Shao *et al.*, 2008; Liu, 2010; Ciarleglio *et al.*, 2015). Most publications fail to define explicitly what exactly constitutes a "success". However, the use of $\text{PoS}'(n)$ implies that rejection of the null hypothesis, irrespective of its truth, must be considered a success. Our analysis confirms the statement in Spiegelhalter *et al.* (2004) that $\text{PoS}'(n)$ can be used as a practical approximation to $\text{PoS}(n)$ in many situations. The exact definition of "probability of success" becomes more interesting when allowing for $\theta_{\text{MCID}} > \theta_0$, a potential extension rarely considered in the literature (see, e.g., Brown *et al.*, 1987, for the binary case).

The exact choice of wording should not be given too much weight. However, we feel that any notion of power in the "hybrid" Bayesian/frequentist setting should be *conditional* on a relevant effect (or at least a non-null effect) to preserve the conditional nature of the purely frequentist power. Using the term "power" to refer to a joint probability like the 'expected power' of Brown *et al.* (1987) and Ciarleglio *et al.* (2015) (our $\text{PoS}(n)$) or the "average/expected power" of Spiegelhalter *et al.* (2004) (our $\text{PoS}'(n)$) is potentially misleading. Others suggest "conditional expected power" for $\text{EP}(n)$ to distinguish it from "expected power" (our $\text{PoS}'(n)$) (Brown *et al.*, 1987; Ciarleglio *et al.*, 2015). This wording, however, may lead to confusion when also considering interim analyses where "conditional power" is a well-established term for the probability of rejecting the null hypothesis given θ_{alt} and partially observed data (Bauer *et al.*, 2016).

A particularly interesting publication is Liu (2010). They extend hybrid sample size derivation in the normal case to also incorporate uncertainty about the variance and clearly distinguish between $\text{PoS}'(n)$ = "extended Bayesian expected power 1", $\text{PoS}(n)$ = "extended Bayesian expected power 2", and

EP(n) = "extended Bayesian expected power 3". Apart from nomenclature, our definitions of these three quantities only differ in that they assume the standard deviation to be fixed and the fact that we accommodate the optional notion of a relevant effect via θ_{MCID}. The former makes explicit formulas more manageable, the latter is important to keep sample sizes small in situations with vague or conservative prior information but substantial relevance thresholds. Liu (2010) and Rufibach *et al.* (2016) are also the only publications we found that study the distribution of the quantities that are averaged over. In Ciarleglio *et al.* (2015), the distinction between all three quantities is also made explicit ("expected power" is our PoS′(n), "prior-adjusted power" is our PoS(n), and "conditional expected power" is our EP(n)).

Table 1: Selected publications on "hybrid" sample size derivation based on error rates.

**Concept**	**References**	**Notes**
**Marginal probability to reject $\mathcal{H}_0$**	Crook and Good (1982)	Termed 'strength'; application in multinomial contingency tables.
	Spiegelhalter and Freedman (1986)	Only implicitly mentioned; discussing close relation to PoS(n), termed 'expected/average power' in Spiegelhalter *et al.* (2004).
	Gillett (1994)	Termed 'average power'; focus on replication.
	O'Hagan and Stevens (2001)	Termed 'assurance' or 'expected power'; different from our notion of expected power which is conditional on a relevant effect, see also (O'Hagan *et al.*, 2005).

Concept	References	Notes
	Chuang-Stein (2006)	Termed ‘average probability of success’; discusses other definitions of ‘success’ based on additional criteria for the observed point estimates; discusses how basing the sample size on relevance arguments alone is theoretically correct but ineffective if evidence for larger effect sizes is available, see also Chuang-Stein *et al.* (2011).
	Grouin *et al.* (2007)	Termed ‘predictive power’ and ‘predictive probability to reject $\mathcal{H}_0$’; review of regulatory aspects, discussion of interval-based sample size calculation, and utility considerations.
	Daimon (2008)	Termed ‘hybrid Neyman–Pearson–Bayesian (hNPB) probability‘; application in non-inferiority setting.
	Shao *et al.* (2008)	Termed ‘adjusted power’; review of regulatory aspects, discussion of interval-based sample size calculation, and utility considerations.
	Liu (2010)	Termed ‘extended Bayesian expected power 1’; extended by treating variance as unknown, also consider PoS(n) and EP(n).
	Lan and Wittes (2012)	Termed ‘average power’; discusses upper limit of ‘average power‘ depending on prior choice and suggest truncated priors which would be very close to conditioning on a relevant effect.

Concept	References	Notes
	Carroll (2013)	Termed ‘assurance’ and ‘probability of success’ (PoS); discusses other definitions of success but all definitions are also exclusively based on *observed* quantities (minimum threshold on point estimate), see also Chuang-Stein (2006).
	Brutti *et al.* (2014)	Termed ‘predictive frequentist power’; also discusses sample size derivation based on Bayesian decision criteria.
	Ren and Oakley (2014)	Termed ‘assurance’; discusses ideas of O’Hagan *et al.* (2005) in time-to-event setting.
	Hu (2014)	Termed ‘probability of success’; considers priors on mean and standard deviation; discuss upper limit on probability of success in the more complex two-parameter situation.
	Ibrahim *et al.* (2015)	Termed ‘average probability of success’; discussed in context of historical data integration.
	Walley *et al.* (2015)	Termed ‘assurance’ or ‘probability of success’; extension to multi-parameter situations.
	Ciarleglio *et al.* (2015)	Termed ‘expected power’; also consider EP(n) and PoS(n), very similar settings considered in Ciarleglio *et al.* (2016); Ciarleglio and Arendt (2017).
	Rufibach *et al.* (2016)	Termed ‘assurance’ or ‘probability of success’; in-depth discussion of the distribution of the probability to reject the null hypothesis.

Concept	References	Notes
	Saint-Hilary *et al.* (2018)	Termed 'predictive probability of success'; consider both 'statistical success' (p-value $\leq \alpha$) and 'clinical relevance' (*observed* effect above relevance threshold), see also Saint-Hilary *et al.* (2019).
	Chen and Ho (2017)	Termed 'assurance' and 'expected power'; discusses conditional nature of the (frequentist) probability to reject the null hypothesis from a Bayesian perspective.
	Jiang (2011); Kirby *et al.* (2012); Zhang and Zhang (2013); Wang *et al.* (2015); Götte *et al.* (2017)	Termed 'probability of statistical success', 'probability of success', 'assurance', 'predictive power'; discusses extensions to multiple studies or entire drug development programs.
	Ambrosius *et al.* (2012); Wang *et al.* (2013); Wang (2015); Crisp *et al.* (2018); Chen and Chen (2018)	Termed 'assurance', 'probability of success', 'probability of study success'; practical applications in various settings.
**Probability of success**	Spiegelhalter and Freedman (1986)	Only implicitly mentioned, termed 'prior adjusted power' in Spiegelhalter *et al.* (2004); discusses close relation to marginal probability to reject $\mathcal{H}_0$ (suggesting the latter as practical approximation).

Concept	References	Notes
	Brown *et al.* (1987)	Termed 'expected power'; also discusses 'conditional expected power' which corresponds to our definition of EP(n).
	Shao *et al.* (2008)	Termed 'adjusted power'; application of the ideas of Spiegelhalter *et al.* (2004) to binary setting, define probability of success but approximate it with the marginal probability to reject $\mathcal{H}_0$.
	Liu (2010)	Termed 'extended Bayesian expected power 2'; extended by treating variance as unknown, also considers PoS$'(n)$ and EP(n).
	Ciarleglio *et al.* (2015)	Termed 'prior-adjusted power'; also considers EP(n) and PoS$'(n)$, very similar settings considered in Ciarleglio *et al.* (2016); Ciarleglio and Arendt (2017).
**Expected power**	Brown *et al.* (1987)	Termed 'conditional expected power'; also discusses unconditional expected power which corresponds to our definition of PoS(n).
	Spiegelhalter *et al.* (2004)	Not named; referencing Brown *et al.* (1987).
	Liu (2010)	Termed 'extended Bayesian expected power 3'; extended by treating variance as unknown, also consider PoS(n) and PoS(n).
	Ciarleglio *et al.* (2015)	Termed 'conditional expected power'; also considers PoS(n) and PoS$'(n)$, very similar settings considered in Ciarleglio *et al.* (2016); Ciarleglio and Arendt (2017).

References

Ambrosius, W. T., Polonsky, T. S., Greenland, P., Goff Jr, D. C., Perdue, L. H., Fortmann, S. P., Margolis, K. L., and Pajewski, N. M. (2012). 'Design of the value of imaging in enhancing the wellness of your heart (view) trial and the impact of uncertainty on power.' *Clinical Trials*, **9**(2), 232–246.

Bauer, P., Bretz, F., Dragalin, V., König, F., and Wassmer, G. (2016). 'Twenty-five years of confirmatory adaptive designs: opportunities and pitfalls.' *Statistics in Medicine*, **35**(3), 325–347.

Brown, B. W., Herson, J., Atkinson, E. N., and Rozell, M. E. (1987). 'Projection from previous studies: a bayesian and frequentist compromise.' *Controlled Clinical Trials*, **8**(1), 29–44.

Brutti, P., De Santis, F., and Gubbiotti, S. (2014). 'Bayesian-frequentist sample size determination: a game of two priors.' *Metron*, **72**(2), 133–151.

Carroll, K. J. (2013). 'Decision making from phase II to phase III and the probability of success: reassured by "assurance"?' *Journal of Biopharmaceutical Statistics*, **23**(5), 1188–1200.

Chen, D.-G. and Chen, J. K. (2018). 'Statistical power and bayesian assurance in clinical trial design.' In 'New Frontiers of Biostatistics and Bioinformatics,' Springer, New York, pages 193–200.

Chen, D.-G. and Ho, S. (2017). 'From statistical power to statistical assurance: It's time for a paradigm change in clinical trial design.' *Communications in Statistics-Simulation and Computation*, **46**(10), 7957–7971.

Chuang-Stein, C. (2006). 'Sample size and the probability of a successful trial.' *Pharmaceutical Statistics*, **5**(4), 305–309.

Chuang-Stein, C., Kirby, S., Hirsch, I., and Atkinson, G. (2011). 'The role of the minimum clinically important difference and its impact on designing a trial.' *Pharmaceutical Statistics*, **10**(3), 250–256.

Ciarleglio, M. M. and Arendt, C. D. (2017). 'Sample size determination for a binary response in a superiority clinical trial using a hybrid classical and Bayesian procedure.' *Trials*, **18**(1), 83.

Ciarleglio, M. M., Arendt, C. D., Makuch, R. W., and Peduzzi, P. N. (2015). 'Selection of the treatment effect for sample size determination in a superiority clinical trial using a hybrid classical and bayesian procedure.' *Contemporary Clinical Trials*, **41**, 160–171.

Ciarleglio, M. M., Arendt, C. D., and Peduzzi, P. N. (2016). 'Selection of the effect size for sample size determination for a continuous response in a superiority clinical trial using a hybrid classical and Bayesian procedure.' *Clinical Trials*, **13**(3), 275–285.

Crisp, A., Miller, S., Thompson, D., and Best, N. (2018). 'Practical experiences of adopting assurance as a quantitative framework to support decision making in drug development.' *Pharmaceutical Statistics*, **17**(4), 317–328.

Crook, J. F. and Good, I. J. (1982). 'The powers and strengths of tests for multinomials and contingency tables.' *Journal of the American Statistical Association*, **77**(380), 793–802.

Daimon, T. (2008). 'Bayesian sample size calculations for a non-inferiority test of two proportions in clinical trials.' *Contemporary Clinical Trials*, **29**(4), 507–516.

Gillett, R. (1994). 'An average power criterion for sample size estimation.' *Journal of the Royal Statistical Society: Series D (The Statistician)*, **43**(3), 389–394.

Götte, H., Kirchner, M., and Sailer, M. O. (2017). 'Probability of success for phase III after exploratory biomarker analysis in phase II.' *Pharmaceutical Statistics*, **16**(3), 178–191.

Grouin, J.-M., Coste, M., Bunouf, P., and Lecoutre, B. (2007). 'Bayesian sample size determination in non-sequential clinical trials: Statistical aspects and some regulatory considerations.' *Statistics in Medicine*, **26**(27), 4914–4924.

Hu, P. (2014). 'Probability of success: estimation framework, properties and applications.' *Stat*, **3**(1), 158–171.

Ibrahim, J. G., Chen, M.-H., Lakshminarayanan, M., Liu, G. F., and Heyse, J. F. (2015). 'Bayesian probability of success for clinical trials using historical data.' *Statistics in Medicine*, **34**(2), 249–264.

Jiang, K. (2011). ‘Optimal sample sizes and go/no-go decisions for phase II/III development programs based on probability of success.’ *Statistics in Biopharmaceutical Research*, **3**(3), 463–475.

Kirby, S., Burke, J., Chuang-Stein, C., and Sin, C. (2012). ‘Discounting phase 2 results when planning phase 3 clinical trials.’ *Pharmaceutical Statistics*, **11**(5), 373–385.

Lan, K. G. and Wittes, J. T. (2012). ‘Some thoughts on sample size: a bayesian-frequentist hybrid approach.’ *Clinical Trials*, **9**(5), 561–569.

Liu, F. (2010). ‘An extension of bayesian expected power and its application in decision making.’ *Journal of Biopharmaceutical Statistics*, **20**(5), 941–953.

O’Hagan, A. and Stevens, J. W. (2001). ‘Bayesian assessment of sample size for clinical trials of cost-effectiveness.’ *Medical Decision Making*, **21**(3), 219–230.

O’Hagan, A., Stevens, J. W., and Campbell, M. J. (2005). ‘Assurance in clinical trial design.’ *Pharmaceutical Statistics*, **4**(3), 187–201.

Ren, S. and Oakley, J. E. (2014). ‘Assurance calculations for planning clinical trials with time-to-event outcomes.’ *Statistics in Medicine*, **33**(1), 31–45.

Rufibach, K., Burger, H., and Abt, M. (2016). ‘Bayesian predictive power: choice of prior and some recommendations for its use as probability of success in drug development.’ *Pharmaceutical Statistics*, **15**(5), 438–446.

Saint-Hilary, G., Barboux, V., Pannaux, M., Gasparini, M., Robert, V., and Mastrantonio, G. (2019). ‘Predictive probability of success using surrogate endpoints.’ *Statistics in Medicine*, **38**(10), 1753–1774.

Saint-Hilary, G., Robert, V., and Gasparini, M. (2018). ‘Decision-making in drug development using a composite definition of success.’ *Pharmaceutical Statistics*, **17**(5), 555–569.

Shao, Y., Mukhi, V., and Goldberg, J. D. (2008). ‘A hybrid bayesian-frequentist approach to evaluate clinical trial designs for tests of superiority and non-inferiority.’ *Statistics in Medicine*, **27**(4), 504–519.

Spiegelhalter, D. J., Abrams, K. R., and Myles, J. P. (2004). *Bayesian approaches to clinical trials and health-care evaluation*, volume 13. John Wiley & Sons, Hoboken, New Jersey.

Spiegelhalter, D. J. and Freedman, L. S. (1986). 'A predictive approach to selecting the size of a clinical trial, based on subjective clinical opinion.' *Statistics in Medicine*, **5**(1), 1–13.

Walley, R. J., Smith, C. L., Gale, J. D., and Woodward, P. (2015). 'Advantages of a wholly bayesian approach to assessing efficacy in early drug development: a case study.' *Pharmaceutical Statistics*, **14**(3), 205–215.

Wang, M., Liu, G. F., and Schindler, J. (2015). 'Evaluation of program success for programs with multiple trials in binary outcomes.' *Pharmaceutical Statistics*, **14**(3), 172–179.

Wang, M.-D. (2015). 'Applications of probability of study success in clinical drug development.' In 'Applied Statistics in Biomedicine and Clinical Trials Design,' Springer, pages 185–196.

Wang, Y., Fu, H., Kulkarni, P., and Kaiser, C. (2013). 'Evaluating and utilizing probability of study success in clinical development.' *Clinical Trials*, **10**(3), 407–413.

Zhang, J. and Zhang, J. J. (2013). 'Joint probability of statistical success of multiple phase III trials.' *Pharmaceutical Statistics*, **12**(6), 358–365.
